# Bis[5-(2-naphth­yl)-1*H*-pyrazole-κ*N* ^2^]silver(I) nitrate

**DOI:** 10.1107/S1600536809028645

**Published:** 2009-07-25

**Authors:** Zhao-Yang Wang, Pei-Pei Zhang, Guang Yang, Seik Weng Ng

**Affiliations:** a Department of Chemistry, Zhengzhou University, Zhengzhou 450001, People’s Republic of China; b Department of Chemistry, University of Malaya, 50603 Kuala Lumpur, Malaysia

## Abstract

The Ag atom in the title compound, [Ag(C_13_H_10_N_2_)_2_]NO_3_, shows an approximately linear coordination [N–Ag–N 162.6 (4)°]. The coordination geometry is distorted towards square-planar owing to two long Ag⋯O inter­actions [Ag⋯O = 2.634 (15) and 2.861 (13) Å]. In the crystal structure, the Ag atom lies on a special position of *2* site symmetry; the nitrate anion is disordered about the special position. The crystal under investigation was a racemic twin with a 33% minor twin component.

## Related literature

This structure is the first report of a metal complex of the 5-(2-naphthyl)-1*H*-pyrazole; for the synthesis of this *N*-heterocycle, see: Yang & Raptis (2003 ▶).
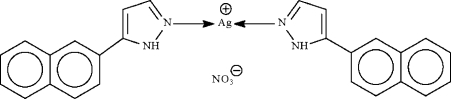


## Experimental

### 

#### Crystal data

[Ag(C_13_H_10_N_2_)_2_]NO_3_
*M* *_r_* = 558.34
Monoclinic, 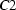

*a* = 13.911 (6) Å
*b* = 7.340 (1) Å
*c* = 12.669 (5) Å
β = 113.43 (2)°
*V* = 1186.9 (7) Å^3^
*Z* = 2
Mo *K*α radiation
μ = 0.89 mm^−1^
*T* = 291 K
0.20 × 0.18 × 0.16 mm

#### Data collection

Rigaku R-AXIS RAPID IP diffractometer
Absorption correction: multi-scan (*ABSCOR*; Higashi, 1995 ▶) *T*_min_ = 0.843, *T*_max_ = 0.871
2268 measured reflections
2056 independent reflections
1752 reflections with *I* > 2σ(*I*)
*R*_int_ = 0.061

#### Refinement

*R*[*F* ^2^ > 2σ(*F* ^2^)] = 0.061
*wR*(*F* ^2^) = 0.174
*S* = 1.09
2056 reflections
179 parameters
32 restraints
H-atom parameters constrained
Δρ_max_ = 0.36 e Å^−3^
Δρ_min_ = −0.48 e Å^−3^
Absolute structure: Flack (1983 ▶), 353 Friedel pairs
Flack parameter: 0.33 (8)

### 

Data collection: *RAPID-AUTO* (Rigaku, 1998 ▶); cell refinement: *RAPID-AUTO*; data reduction: *CrystalStructure* (Rigaku/MSC, 2002 ▶); program(s) used to solve structure: *SHELXS97* (Sheldrick, 2008 ▶); program(s) used to refine structure: *SHELXL97* (Sheldrick, 2008 ▶); molecular graphics: *X-SEED* (Barbour, 2001 ▶); software used to prepare material for publication: *publCIF* (Westrip, 2009 ▶).

## Supplementary Material

Crystal structure: contains datablocks I, global. DOI: 10.1107/S1600536809028645/xu2561sup1.cif

Structure factors: contains datablocks I. DOI: 10.1107/S1600536809028645/xu2561Isup2.hkl

Additional supplementary materials:  crystallographic information; 3D view; checkCIF report

## Figures and Tables

**Fig. 1.**
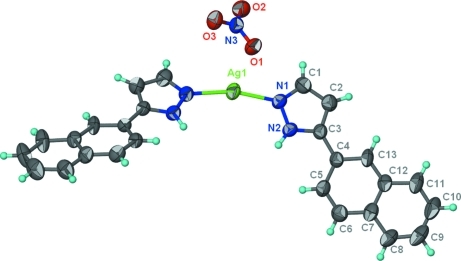
Thermal ellipsoid plot of the [Ag(C13H10N2)2][NO3], displacement ellipsoids are drawn at the 50% probability level. The nitrate anion is disordered about a special position; the disorder is not shown.

## supplementary crystallographic information

### Experimental

3-(2-Naphthyl)pyrazole was prepared according to the literature method (Yang & Raptis, 2003). An acetonitrile solution (2 ml) of silver nitrate (0.02 mmol, 3.4 mg) was mixed with an ethanol solution (1 ml) of 3-(2-naphthyl)pyrazole (0.02 mmol, 4 mg). The pH value of the mixture was adjusted to about 5 by dilute nitric acid. The resulting solution was allowed to evaporate for two weeks to yield colorless crystals in 60% yield.

### Refinement

The measurements are complete to 94% at a 2θ of 55 °, but are complete to 98% at a 2θ of 50 °.

The nitrate group is disordered about the twofold rotation axis; the anion was allowed to refine off the symmetry element. The three N–O distances were restrained to within 0.01 Å of each other, as were the three O···O distances. The four atoms were restrained to be nearly planar, and their anisotropic temperature factors were restrained to be nearly isotropic. Carbon-bound H atoms were placed in calculated positions [C—H = 0.93, N–H 0.89 Å; *U*(H) = 1.2*U*_eq_(*C*,*N*)].

The crystal under investigation is a racemic twin; the explicit refinement of the Flack parameter gave a minor twin component of 33%.

### Crystal data

**Table:**

[Ag(C_13_H_10_N_2_)_2_]NO_3_	*F*(000) = 564
*M**_r_* = 558.34	*D*_x_ = 1.562 Mg m^−^^3^
Monoclinic, *C*2	Mo *K*α radiation, λ = 0.71073 Å
Hall symbol: C 2y	Cell parameters from 2056 reflections
*a* = 13.911 (6) Å	θ = 1.8–27.5°
*b* = 7.340 (1) Å	µ = 0.89 mm^−^^1^
*c* = 12.669 (5) Å	*T* = 291 K
β = 113.43 (2)°	Block, colorless
*V* = 1186.9 (7) Å^3^	0.20 × 0.18 × 0.16 mm
*Z* = 2	

### Data collection

**Table:**

Rigaku R-AXIS RAPID IP diffractometer	2056 independent reflections
Radiation source: fine-focus sealed tube	1752 reflections with *I* > 2σ(*I*)
graphite	*R*_int_ = 0.061
ω scans	θ_max_ = 27.5°, θ_min_ = 1.8°
Absorption correction: multi-scan (*ABSCOR*; Higashi, 1995)	*h* = −17→8
*T*_min_ = 0.843, *T*_max_ = 0.871	*k* = −9→7
2268 measured reflections	*l* = −14→16

### Refinement

**Table:**

Refinement on *F*^2^	Hydrogen site location: inferred from neighbouring sites
Least-squares matrix: full	H-atom parameters constrained
*R*[*F*^2^ > 2σ(*F*^2^)] = 0.061	*w* = 1/[σ^2^(*F*_o_^2^) + (0.1009*P*)^2^ + 2.0218*P*] where *P* = (*F*_o_^2^ + 2*F*_c_^2^)/3
*wR*(*F*^2^) = 0.174	(Δ/σ)_max_ = 0.001
*S* = 1.09	Δρ_max_ = 0.36 e Å^−^^3^
2056 reflections	Δρ_min_ = −0.48 e Å^−^^3^
179 parameters	Extinction correction: *SHELXL97* (Sheldrick, 2008), Fc^*^=kFc[1+0.001xFc^2^λ^3^/sin(2θ)]^-1/4^
32 restraints	Extinction coefficient: 0.010 (2)
Primary atom site location: structure-invariant direct methods	Absolute structure: Flack (1983), 353 Friedel pairs
Secondary atom site location: difference Fourier map	Flack parameter: 0.33 (8)

### Fractional atomic coordinates and isotropic or equivalent isotropic displacement parameters (Å^2^)

**Table:**

	*x*	*y*	*z*	*U*_iso_*/*U*_eq_	Occ. (<1)
Ag1	0.5000	0.5000 (2)	0.5000	0.0696 (4)	
O1	0.5305 (13)	0.8398 (18)	0.4537 (12)	0.079 (4)	0.50
O2	0.4805 (13)	1.1184 (16)	0.4497 (12)	0.078 (4)	0.50
O3	0.4805 (13)	0.9378 (17)	0.5844 (11)	0.079 (4)	0.50
N1	0.6133 (6)	0.4563 (10)	0.4293 (6)	0.060 (2)	
N2	0.6235 (6)	0.2913 (10)	0.3832 (6)	0.0565 (16)	
H2	0.5892	0.1907	0.3868	0.068*	
N3	0.4971 (9)	0.9654 (11)	0.4961 (9)	0.062 (3)	0.50
C1	0.6779 (7)	0.5684 (15)	0.4085 (8)	0.068 (2)	
H1	0.6879	0.6898	0.4312	0.082*	
C2	0.7299 (7)	0.479 (3)	0.3471 (7)	0.068 (3)	
H2A	0.7788	0.5297	0.3225	0.082*	
C3	0.6928 (7)	0.3009 (12)	0.3311 (7)	0.0546 (18)	
C4	0.7151 (6)	0.1443 (12)	0.2722 (7)	0.0537 (18)	
C5	0.6475 (6)	−0.008 (3)	0.2316 (6)	0.0616 (18)	
H5	0.5874	−0.0157	0.2467	0.074*	
C6	0.6685 (8)	−0.1416 (16)	0.1720 (8)	0.070 (2)	
H6	0.6211	−0.2367	0.1431	0.084*	
C7	0.7620 (8)	−0.1405 (17)	0.1522 (8)	0.067 (2)	
C8	0.7883 (9)	−0.282 (2)	0.0913 (9)	0.080 (3)	
H8	0.7440	−0.3821	0.0639	0.096*	
C9	0.8788 (10)	−0.271 (2)	0.0733 (9)	0.096 (4)	
H9	0.8948	−0.3637	0.0328	0.115*	
C10	0.9456 (8)	−0.130 (3)	0.1125 (10)	0.094 (4)	
H10	1.0069	−0.1275	0.0997	0.113*	
C11	0.9243 (6)	0.009 (4)	0.1703 (6)	0.082 (3)	
H11	0.9719	0.1039	0.1966	0.098*	
C12	0.8311 (6)	0.014 (3)	0.1924 (6)	0.060 (2)	
C13	0.8064 (6)	0.1497 (16)	0.2520 (8)	0.063 (2)	
H13	0.8519	0.2479	0.2795	0.075*	

### Atomic displacement parameters (Å^2^)

**Table:**

	*U*^11^	*U*^22^	*U*^33^	*U*^12^	*U*^13^	*U*^23^
Ag1	0.0884 (6)	0.0661 (6)	0.0753 (6)	0.000	0.0547 (5)	0.000
O1	0.077 (7)	0.070 (8)	0.097 (8)	0.012 (6)	0.043 (6)	−0.013 (7)
O2	0.089 (8)	0.058 (7)	0.093 (8)	0.009 (6)	0.043 (7)	0.006 (6)
O3	0.085 (7)	0.088 (9)	0.076 (7)	0.007 (6)	0.045 (6)	0.005 (6)
N1	0.076 (4)	0.052 (6)	0.062 (4)	0.002 (3)	0.039 (3)	−0.003 (3)
N2	0.072 (4)	0.050 (4)	0.056 (4)	−0.001 (3)	0.034 (4)	−0.004 (3)
N3	0.054 (4)	0.071 (9)	0.067 (5)	0.001 (10)	0.029 (4)	−0.005 (10)
C1	0.071 (5)	0.074 (6)	0.060 (5)	−0.009 (4)	0.027 (4)	−0.003 (4)
C2	0.068 (4)	0.072 (9)	0.068 (4)	−0.003 (6)	0.033 (3)	0.001 (6)
C3	0.060 (4)	0.057 (4)	0.054 (4)	0.001 (3)	0.029 (4)	0.004 (3)
C4	0.055 (4)	0.053 (4)	0.058 (5)	0.005 (3)	0.028 (4)	0.009 (4)
C5	0.071 (4)	0.060 (5)	0.067 (4)	0.008 (7)	0.041 (3)	0.006 (8)
C6	0.075 (5)	0.074 (6)	0.064 (6)	−0.004 (5)	0.031 (5)	0.002 (5)
C7	0.077 (5)	0.085 (7)	0.048 (5)	0.008 (5)	0.034 (4)	0.004 (4)
C8	0.090 (6)	0.089 (8)	0.068 (5)	0.011 (6)	0.037 (5)	−0.017 (6)
C9	0.108 (9)	0.124 (11)	0.065 (6)	0.030 (8)	0.043 (6)	−0.015 (7)
C10	0.053 (5)	0.160 (14)	0.066 (6)	0.022 (6)	0.019 (5)	−0.023 (7)
C11	0.053 (4)	0.141 (10)	0.055 (4)	0.004 (10)	0.024 (3)	−0.018 (11)
C12	0.056 (3)	0.081 (7)	0.044 (3)	0.002 (7)	0.021 (3)	0.003 (7)
C13	0.051 (4)	0.091 (7)	0.056 (5)	−0.001 (4)	0.032 (4)	−0.004 (4)

### Geometric parameters (Å, °)

**Table:**

Ag1—N1^i^	2.124 (7)	C4—C5	1.42 (2)
Ag1—N1	2.124 (7)	C5—C6	1.34 (2)
Ag1—O1	2.634 (15)	C5—H5	0.9300
Ag1—O2^ii^	2.861 (13)	C6—C7	1.419 (13)
O1—N3	1.246 (10)	C6—H6	0.9300
O2—N3	1.245 (10)	C7—C8	1.427 (15)
O3—N3	1.246 (10)	C7—C12	1.44 (2)
N1—C1	1.319 (12)	C8—C9	1.369 (17)
N1—N2	1.375 (10)	C8—H8	0.9300
N2—C3	1.370 (11)	C9—C10	1.35 (2)
N2—H2	0.8900	C9—H9	0.9300
C1—C2	1.416 (16)	C10—C11	1.35 (3)
C1—H1	0.9300	C10—H10	0.9300
C2—C3	1.39 (2)	C11—C12	1.432 (10)
C2—H2A	0.9300	C11—H11	0.9300
C3—C4	1.469 (12)	C12—C13	1.38 (2)
C4—C13	1.393 (11)	C13—H13	0.9300
			
N1^i^—Ag1—N1	162.6 (4)	C6—C5—C4	121.3 (7)
N1^i^—Ag1—O1	116.7 (4)	C6—C5—H5	119.4
N1—Ag1—O1	80.6 (4)	C4—C5—H5	119.4
N1^i^—Ag1—O2^ii^	85.7 (4)	C5—C6—C7	121.2 (10)
N1—Ag1—O2^ii^	77.2 (4)	C5—C6—H6	119.4
O1—Ag1—O2^ii^	152.4 (5)	C7—C6—H6	119.4
N3—O1—Ag1	119.0 (7)	C6—C7—C8	122.8 (11)
C1—N1—N2	105.7 (7)	C6—C7—C12	118.1 (10)
C1—N1—Ag1	131.9 (7)	C8—C7—C12	119.1 (9)
N2—N1—Ag1	122.0 (5)	C9—C8—C7	119.8 (12)
C3—N2—N1	111.7 (7)	C9—C8—H8	120.1
C3—N2—H2	124.1	C7—C8—H8	120.1
N1—N2—H2	124.1	C10—C9—C8	122.0 (11)
O2—N3—O1	119.7 (7)	C10—C9—H9	119.0
O2—N3—O3	120.3 (7)	C8—C9—H9	119.0
O1—N3—O3	120.0 (7)	C11—C10—C9	120.7 (11)
N1—C1—C2	111.1 (12)	C11—C10—H10	119.6
N1—C1—H1	124.5	C9—C10—H10	119.6
C2—C1—H1	124.5	C10—C11—C12	122 (2)
C3—C2—C1	105.8 (9)	C10—C11—H11	118.8
C3—C2—H2A	127.1	C12—C11—H11	118.8
C1—C2—H2A	127.1	C13—C12—C11	124.7 (18)
C2—C3—N2	105.6 (7)	C13—C12—C7	119.2 (7)
C2—C3—C4	132.0 (7)	C11—C12—C7	116.1 (17)
N2—C3—C4	122.4 (8)	C12—C13—C4	121.7 (10)
C13—C4—C5	118.4 (8)	C12—C13—H13	119.2
C13—C4—C3	117.6 (8)	C4—C13—H13	119.2
C5—C4—C3	123.9 (8)		
			
N1^i^—Ag1—O1—N3	15.5 (10)	C2—C3—C4—C5	−158.6 (11)
N1—Ag1—O1—N3	−166.2 (9)	N2—C3—C4—C5	20.6 (14)
O2^ii^—Ag1—O1—N3	157.0 (11)	C13—C4—C5—C6	−2.1 (16)
N1^i^—Ag1—N1—C1	−170.0 (8)	C3—C4—C5—C6	175.7 (10)
O1—Ag1—N1—C1	15.2 (9)	C4—C5—C6—C7	3.3 (17)
O2^ii^—Ag1—N1—C1	178.7 (9)	C5—C6—C7—C8	178.5 (11)
N1^i^—Ag1—N1—N2	17.3 (6)	C5—C6—C7—C12	−3.4 (15)
O1—Ag1—N1—N2	−157.4 (8)	C6—C7—C8—C9	178.8 (10)
O2^ii^—Ag1—N1—N2	6.0 (6)	C12—C7—C8—C9	0.8 (16)
C1—N1—N2—C3	−1.4 (10)	C7—C8—C9—C10	0.7 (19)
Ag1—N1—N2—C3	173.0 (5)	C8—C9—C10—C11	−1(2)
Ag1—O1—N3—O2	−157.6 (9)	C9—C10—C11—C12	0(2)
Ag1—O1—N3—O3	22.4 (10)	C10—C11—C12—C13	179.1 (13)
N2—N1—C1—C2	0.9 (10)	C10—C11—C12—C7	2(2)
Ag1—N1—C1—C2	−172.6 (6)	C6—C7—C12—C13	2.5 (16)
N1—C1—C2—C3	−0.2 (11)	C8—C7—C12—C13	−179.5 (11)
C1—C2—C3—N2	−0.7 (10)	C6—C7—C12—C11	−179.9 (11)
C1—C2—C3—C4	178.6 (9)	C8—C7—C12—C11	−1.8 (16)
N1—N2—C3—C2	1.3 (10)	C11—C12—C13—C4	−178.8 (12)
N1—N2—C3—C4	−178.1 (8)	C7—C12—C13—C4	−1.4 (17)
C2—C3—C4—C13	19.2 (15)	C5—C4—C13—C12	1.2 (15)
N2—C3—C4—C13	−161.6 (8)	C3—C4—C13—C12	−176.7 (10)

Symmetry codes: (i) −*x*+1, *y*, −*z*+1; (ii) *x*, *y*−1, *z*.

### Hydrogen-bond geometry (Å, °)

**Table:**

*D*—H···*A*	*D*—H	H···*A*	*D*···*A*	*D*—H···*A*
N2—H2···O2^ii^	0.89	2.04	2.76 (2)	137
N2—H2···O3^iii^	0.89	2.19	3.08 (2)	173

Symmetry codes: (ii) *x*, *y*−1, *z*; (iii) −*x*+1, *y*−1, −*z*+1.
